# Interferon-β Modulates Early Viral Replication Kinetics and Innate Responses to Non-Fatal Alphavirus Encephalomyelitis

**DOI:** 10.3390/pathogens15040441

**Published:** 2026-04-18

**Authors:** Benjamin H. Nguyen, Elise Stanley, Victoria K. Baxter, Diane E. Griffin

**Affiliations:** 1 W. Harry Feinstone Department of Molecular Microbiology and Immunology, Johns Hopkins Bloomberg School of Public Health, Baltimore, MD 21205, USA; 2 Texas Biomedical Research Institute, San Antonio, TX 78227, USA; vbaxter@txbiomed.org

**Keywords:** alphavirus, encephalomyelitis, interferon, monocyte, CNS

## Abstract

Alphaviruses are mosquito-borne viruses that can infect the central nervous system (CNS) and cause encephalomyelitis, which is a rare but dangerous complication from infection. In mice, this can be studied in a model of infection with Sindbis virus (SINV), which infects neurons and causes neurological disease. Due to the non-renewable nature of neurons, the immune response in the CNS is specialized to prevent neuronal damage or death, even if they are infected. Therefore, insights into the nuances of antiviral immunity in the CNS provide a better understanding of disease pathogenesis and mechanisms of recovery. Type I interferons (IFNs) are critically important for survival; they are an innate antiviral defense mechanism that consists mainly of IFNα and IFNβ. Although both use the same receptor, type-specific differences between IFNα and IFNβ have been described in other contexts. To this end, *Ifnb*^−/−^ mice were used to elucidate the role of IFNβ in recovery from alphavirus encephalomyelitis. IFNβ-deficient mice have intact IFNα expression and downstream signaling, but symptomatic disease occurs earlier and is more severe. This is accompanied by increased virus replication in the early stages of infection. Microgliosis is reduced in *Ifnb*^−/−^ mice compared to wildtype, but inflammatory cytokine/chemokine levels are higher and associated with alterations in monocyte and NK cell recruitment into the CNS. *Ifnb*^−/−^ mice have no deficiencies in the expression of factors known to be required for viral clearance. Therefore, IFNβ modulates the early stages of the immune response and facilitates restriction of virus replication, contributing to delayed disease onset.

## 1. Introduction

Alphaviruses are a genus of arthropod-borne viruses that periodically cause epidemic febrile, arthritic, and encephalitic diseases. While these viruses have been historically classified as Old World and New World based on geographic distribution and differences in clinical disease, these distinctions are now blurred due to climate change-driven expansion of their mosquito vectors [1,2,3,4]. Neurologic disease is typically associated with the New World alphaviruses, whereas the Old World viruses generally cause rash and arthritis. However, as these viruses spread and replicate in new areas, some Old World viruses gained the ability to cause encephalitis, with chikungunya virus (CHIKV) being the most notable example [5,6,7]. Alphavirus infection of the central nervous system (CNS) causes an inflammatory immune response that can trigger encephalomyelitis, a rare but dangerous complication from infection that can leave survivors with long-term sequela. This can be modeled via infection with Sindbis virus (SINV), an Old World alphavirus that infects neurons in mice and causes encephalomyelitis [8,9,10,11]. Disease outcome is dependent on both host and viral genetics [9,12], and models of recovery use the TE strain of SINV [13], which causes mild disease from which C57BL/6J [14] mice can recover [15,16,17,18,19]. No approved treatments or vaccines exist for alphavirus-induced encephalitic disease; therefore, studies examining the immune determinants of survival and disease severity can aid in the development of targeted medical countermeasures.

Type I interferon (IFN) is an innate, fast-acting antiviral defense mechanism that is critical for host survival from many viral infections [19,20,21]. IFNα and IFNβ are the main players, and the family also includes IFN-ε, -κ, -τ, -δ, -ζ, and -ω, but their roles in antiviral mechanisms are not well defined [22,23]. Type I IFN expression is triggered by pattern recognition receptor (PRR) engagement following viral infection of a cell. Phosphorylation of IFN regulatory factor (IRF) 3 happens first downstream of PRR signaling. IRF3 is constitutively expressed in cells, and its activation induces IFNβ. Like all other members of the type I IFN family, IFNβ is secreted, acts in both autocrine or paracrine manners, and signals through the type I IFN receptor, IFNAR. Through a JAK/STAT pathway, IFNAR signaling triggers the induction of IFN-stimulated genes (ISG) [22,23,24]. There are hundreds of genes considered to be ISGs, which generally have anti-pathogenic effects. One important gene induced by type I IFN is IRF7, which is the master regulator of IFNα [25]. IRF7 expression downstream of IFNβ signaling is required for the production of IFNα [24,25]. Plasmacytoid dendritic cells (pDC) are the exception to this and are considered professional IFNα-expressing cells due to constitutive expression of IRF7 [22,23]. Generally, type I IFN is the first responder to viral infection; it kickstarts the immune response, restricts virus replication, and puts uninfected cells into an antiviral state.

The ISGs induced by type I IFN encompass a wide range of functions, including host defense, inflammation, and transcription. Additionally, some genes act against a wide range of viruses, while others are narrower in scope [26,27,28]. Apart from the induction of antiviral ISGs, which is important at the cellular level, type I IFNs can also act systemically, and they are important regulators of the innate immune response [28]. Type I IFN promotes the expression of chemokines that recruit inflammatory monocytes and NK cells to the site of infection, and this has been demonstrated for multiple viruses [29,30]. In addition to recruitment, type I IFN stimulates NK cell cytotoxicity and IFNγ production [31]. This effect can act directly on NK cells or indirectly through activation of other cells that subsequently release NK cell-activating factors. One such example is type I IFN activation of dendritic cells (DC), which respond directly by upregulating MHC and costimulatory molecules [32,33], and also by the release of cytokines that activate NK cells [34,35,36]. Further, type I IFN can also influence immune cell development [30]. Therefore, the effects of type I IFN are wide-ranging and go beyond restriction of virus replication within infected cells. In any case, acute type I IFN production is beneficial for promoting antiviral responses, but chronic INF production can be harmful [23,28].

There has recently been a greater understanding and appreciation for the negative effects of type I IFN, particularly with chronic expression. Despite the aforementioned activation of the innate immune response, type I IFN also paradoxically inhibits the functions that it enables [28,37]. It can exert anti-proliferative effects on immune cells, suppress macrophage function, and block IFNγ production by NK cells [38,39,40,41]. Further, type I IFN can exacerbate damaging inflammatory responses that result in collateral tissue damage. It is well documented that type I IFN is associated with increased expression of inflammatory cytokines [37], and excess production can result in immunopathology [37,42]. Type I IFN has also been shown to impair tissue repair, particularly of the lungs during respiratory virus infection [43]. However, studies examining the dual nature of type I IFN are highly variable and context specific—it can be both deleterious and cytoprotective when ablated, depending on the situation. Within the CNS specifically, low levels of type I IFN expression prime the immune system for rapid response to injury or threat [44,45,46], and under homeostatic conditions, type I IFN contributes to neuronal proteostasis, synaptic plasticity, and cognitive capacity [44]. Therefore, alterations to type I IFN levels during viral infection could contribute toward clinical symptoms via disruption of type I IFN-mediated maintenance of neuronal function.

Interestingly, type-specific functions for IFNα and IFNβ have been described despite their usage of the same receptor [47,48,49,50,51]. For example, IFNα restricts virus replication and limits spread, while IFNβ regulates neutrophil recruitment during peripheral infection with CHIKV, a related alphavirus [49,50]. Similarly, selective blockade of IFNα affected initial lymphatic choriomeningitis virus dissemination, while blockade of IFNβ affected lymphocyte responses [48]. IFNβ is more potent than IFNα for controlling West Nile virus infection [20,51], and the different IFNα subtypes have varying effects on hepatitis B virus infection [52]. Current data support the notion that binding affinity determines signaling and function, with IFNβ having the highest natural affinity for the receptor [53,54,55]. In the context of SINV infection of the CNS, type I IFN is critically important for survival, as IFNAR knockout mice die from infection [19], but the specific roles of IFNα and IFNβ have not been examined aside from one study looking at the effect of IFNβ deficiency on SINV titers in the CNS [56].

Taken together, type I IFN has an extremely broad range of functions that are both type- and context-specific. Therefore, we sought to determine the effects of IFNβ on disease outcome and immune responses during SINV-induced encephalomyelitis. These studies focus on the early stages of infection and examine the effects of IFNβ on virus replication and innate immunity. *Ifnb*^−/−^ mice have altered clinical disease that is associated with higher virus replication at the time of disease onset and alterations to the inflammatory response, with lower gliosis but higher chemokine production. The composition of immune cells infiltrating into the CNS during infection was also altered in the absence of IFNβ. Thus, IFNβ exerts both antiviral and immunomodulatory functions that alter disease outcome from SINV infection.

## 2. Methods

### 2.1. Sindbis Virus Infection of Mice

Wildtype [14] C57BL/6J mice were obtained from Jackson Laboratories. *Ifnb*^−/−^ mice [57] were a gift from Deborah Lenschow and Michael Diamond (Washington University, St. Louis, MO, USA). All mice were bred in-house, and transgenic mice were on a WT C57BL/6J background.

The SINV TE strain [13] was grown on BHK-21 cells and quantified by plaque formation. For infection, 5–8-week-old mice of both sexes were briefly anesthetized with isoflurane and intracranially inoculated in the left cerebral hemisphere with 10^3^ PFU of virus diluted in 20 µL of phosphate-buffered saline (PBS).

For clinical evaluation, mice were weighed daily and scored for signs of neurologic disease using the following 6-point system: 0 = clinically normal; 1 = abnormal gait and/or ataxia; 2 = mild hunched posture with occasional two-leg stance but normal demeaner and ambulation; 3 = markedly hunched posture with minimal ambulation or obtunded; 4 = unilateral hindlimb paralysis; 5 = bilateral hindlimb paralysis or moribund; and 6 = death. All experiments were performed in accordance with protocols approved by the institutional animal care and use committee of Johns Hopkins University.

### 2.2. Tissue Collection

Mice were euthanized with isoflurane and perfused with PBS. Brains and spinal cords were collected, flash-frozen, and stored at −80 °C. For tissues processed for histology, PBS perfusion was followed by perfusion with 4% paraformaldehyde (PFA) [58], and the collected tissues were fixed in 4% PFA overnight at 4 °C. Tissues were then washed multiple times with PBS, and spinal cords were decalcified using 10% sodium citrate and 22% formic acid on a rotor for 24 h. Fixed tissues were embedded in paraffin and sectioned by the Johns Hopkins University School of Medicine Reference Histology Laboratory.

### 2.3. Enzyme-Linked Immunosorbent Assay (ELISA)

For total protein extraction, previously frozen tissues were homogenized at 20% weight/volume (*w*/*v*; brains) or 10% *w*/*v* (spinal cords) in PBS containing cOmplete Protease Inhibitor Cocktail (Roche, Basel, Switzerland) and PhosSTOP phosphatase inhibitor (Roche) using Lysing Matrix A tubes (MP Biomedicals, Irvine, CA, USA) for 40 s at 6.0 M/s in a FastPrep-24 homogenizer (MP Biomedicals). Commercial ELISA kits were used to measure IFNα (PBL Assay Sciences, Piscataway, NJ, USA), IFNβ (PBL Assay Sciences), and IFNγ (BioLegend, San Diego, CA, USA) levels according to the manufacturer’s instructions. Samples were originally tested at a 1:2 dilution in duplicate and re-run with further dilution if protein levels exceeded the upper limit of detection of the assay.

Anti-SINV antibody was measured with a custom ELISA. Maxisorp 96-well plates (Thermo Scientific, Waltham, MA, USA) were coated with 10^6^ PFU of SINV overnight at 4 °C and subsequently blocked with 10% FBS in PBS + 0.05% Tween-20 (PBS-T) for 2 h at 37 °C. Samples were diluted 1:3 in PBS-T + 10% FBS and incubated overnight at 4 °C. Plates were incubated with HRP-conjugated goat anti-mouse IgM or IgG (Southern Biotech, Birmingham, AL, USA) diluted 1:1000 in PBS-T + 10% FBS for 2 h at RT and developed using the OptEIA TMB Substrate Reagent kit (BD Pharmingen, San Diego, CA, USA) with 2 M H_2_SO_4_ as the stop solution. Washes between every step were in PBS-T. Plates were read at 450 nm, and optical density (OD) values for blank wells were subtracted from OD values of each sample.

### 2.4. Real Time Quantitative Reverse Transcription PCR (qRT-PCR)

For total RNA isolation, previously frozen tissues were homogenized in QIAzol (Qiagen, Hilden, Germany) using Lysing Matrix D tubes (MP Biomedicals) for 40 s at 6.0 M/s in a FastPrep-24 homogenizer (MP Biomedicals). RNA was extracted with chloroform, precipitated with isopropanol, cleaned up with the RNeasy Plus Mini kit (Qiagen), and quantified with a nanodrop spectrophotometer. cDNA was synthesized using the SuperScript III First-Strand Synthesis System (Invitrogen, Carlsbad, CA, USA) with normalized RNA concentrations across samples and oligo dT primers according to the manufacturer’s instructions. The cDNA was diluted, and qRT-PCR was performed using PrimeTime gene expression assays (Integrated DNA Technologies, Coralville, IA, USA) and EagleTaq Universal Master Mix (Roche). *Gapdh* mRNA levels were determined using the TaqMan Rodent GAPDH Control Reagents (Applied Biosystems, Carlsbad, CA, USA). The target gene Ct value was normalized to the Ct value of *Gapdh*. This normalized value was used to calculate the gene expression level relative to the average of the uninfected control value.

SINV RNA quantification was performed using the following primers and probe specific for SINV *nsP2*: primer nsP2 3373F (5′-CCG CAA GTA TGG GTA CGA TCA-3′), primer nsP2 3454R (5′-GTG CCC TTC CCA GCT AGC T-3′), and TaqMan probe nsP2 3317 (5′–6-carboxyfluorescein [6-FAM]–CCA TTG CCG CCG AAC TCT CCC–6-carboxytetramethylrhodamine [6-TAMRA]–3′). RNA copy numbers were quantified using a standard curve consisting of 10-fold serial dilutions ranging from 3 × 10^7^ to 300 copies of the pCRII-TOPO plasmid containing the SINV nsP2 region and normalized to those for *Gapdh*. All reactions were run on an Applied Biosystems 7500 real-time PCR machine.

### 2.5. Virus Quantification

For quantification of infectious virus in CNS tissue by plaque formation, protein homogenates were collected as described and serially diluted in Dulbecco’s modified Eagle’s medium (Gibco, Waltham, MA, USA) + 1% heat-inactivated fetal bovine serum (FBS; Gibco). Samples were incubated on BHK-21 cells for 1 h at 37 °C with shaking. Cells were overlaid with agarose containing 2X minimal essential media (Gibco) + 2% FBS, incubated at 37 °C, 5% CO_2_ for 48 h, and stained with 10% neutral red (Sigma Aldrich, Saint Louis, MO, USA). Plaques were counted and presented as log_10_PFU per gram of tissue.

### 2.6. Histology

Sagittal brain sections (1–2 section replicates per slide) were stained with hematoxylin and eosin (H&E), blinded, scanned at 20× magnification on a Zeiss Axio Scan.Z1 (Zeiss, Oberkochen, Germany), and scored using a modified version of our previously described [15] 4-point system: 0 = no detectable inflammation; 1 = less than 5 inflammatory foci per section; 2 = moderate inflammatory foci in up to 50% of 4X fields; and 3 = moderate to large inflammatory foci in greater than 50% of 4X fields. An additional point was added for excessive parenchymal cellularity, allowing for a maximal score of 4. Copies of some original photographs and photomicrographs were cropped and/or adjusted globally for white balance or brightness using Concentriq software (version 4.5, Proscia, Philadelphia, PA, USA).

Transverse sections of cervical, thoracic, and lumbar spinal cords (three replicates of each section per slide) were stained with H&E, blinded, scanned at 20× magnification on a Zeiss Axio Scan.Z1, and scored using a 3-point scale adapted from the brain scoring system [15]: 0 = no detectable inflammation; 1 = one to two small inflammatory foci per section; and 2 = greater than two inflammatory foci per spinal cord or spinal cords with moderate to marked inflammatory foci. An additional point was added for excessive parenchymal cellularity, allowing for a maximal score of 3. Copies of some original photographs and photomicrographs were cropped and/or adjusted globally for white balance or brightness using Concentriq software.

### 2.7. Western Blotting

Tissue homogenates were collected as described, and protein concentrations were determined using a DC assay kit (Bio-Rad, Hercules, CA, USA). Samples were diluted to equal concentrations in radioimmunoprecipitation assay (RIPA) buffer (50 mM Tris-Cl [pH 8.0], 150 mM NaCl, 1% NP-40, 0.1% SDS, 0.5% Na-deoxycholate, 1 mM EDTA). Proteins were separated by SDS-PAGE, transferred to a nitrocellulose membrane (Bio-Rad), and blocked for 1 h at RT in Tris-buffered saline (TBS) + 0.1% Tween-20 (TBS-T) + 5% milk powder. The following antibodies were diluted in TBS-T + 5% bovine serum albumin (BSA, Sigma, Saint Louis, MO, USA) and incubated overnight at 4 °C with shaking: anti-GFAP (1:10,000; EMD Millipore, Burlington, MA, USA), anti-IBA1 (1:1000; Wako Chemical Laboratories, Richmond, VA, USA), and anti-actin (1:5000; EMD Millipore). Horseradish peroxidase (HRP)-conjugated anti-rabbit or -mouse IgG (Cell Signaling Technologies, Danvers, MA, USA) was diluted 1:1000 in TBS-T + 1% milk and incubated for 1 h at RT with shaking. Washes between every step were in TBS-T. The membranes were developed using the Amersham ECL Prime Western blotting detection reagent (GE Healthcare, Chicago, IL, USA) and visualized using a ChemiDoc MP Imaging System (Bio-Rad). ImageJ (version 1.53k) was used for densitometry analysis; bands of interest were normalized to actin bands within each sample and values represent the immunoreactivity of the protein of interest relative to uninfected WT mice.

### 2.8. Mononuclear Cell Isolation

Single-cell suspensions were made from freshly isolated brains and spinal cords by homogenizing three times in RPMI containing 1% FBS, 1 mg/mL collagenase IV (Worthington Biochemical Corporation, Lakewood, NJ, USA), and 0.1 mg/mL DNase I (Stem Cell Technologies, Vancouver, BC, Canada) using the GentleMACS system (Miltenyi, Bergisch Gladbach, Germany), with a 15 min 37 °C incubation and gentle agitation between each round of homogenization. Homogenates were filtered through 70 µm filters and washed with RPMI containing 1% FBS. Myelin debris and erythrocytes were removed by centrifugation on a 30/70% percoll gradient for 30 min at 4 °C. Mononuclear cells were collected at the interface, erythrocytes lysed with ACK lysing buffer (Quality Biological, Gaithersburg, MD, USA), and cells were washed several times with RPMI containing 1% FBS before freezing in a 10% DMSO and 90% FBS solution.

### 2.9. Flow Cytometry

After mononuclear cell isolation, cells were thawed, washed, counted, and 10^6^ cells were plated to be stained with the LIVE/DEAD Fixable Near-IR 633 dead cell stain (Invitrogen) in PE buffer (1× PBS, 2 mM EDTA), blocked with rat anti-mouse CD16/CD32 (BD Pharmingen, clone 2.4G2) diluted in PE buffer, and surface stained with an antibody cocktail in FACS buffer (1× PBS, 2 mM EDTA, 0.5% BSA) for 30 min at RT. The following antibodies from BioLegend (San Diego, CA, USA) or BD Pharmingen were used for surface staining (Table S2): CD45 (clone 30-F11), CD11b (clone M1/70), F4/80 (clone QA17A29), I-A/I-E (MHC-II; clone M5/114.15.2), Ly6C (clone HK1.4), CD43 (clone S7), CD11c (clone N418), CD103 (clone 2E7), SiglecH (clone 551), CD3 (clone 17A2), CD4 (clone GK1.5), CD8a (clone 53-6.7), NK1.1 (clone S17016D), CD19 (clone 6D5), and Ly6G (clone 1A8).

Following staining, cells were washed, suspended in FACS buffer, and data were acquired in the Johns Hopkins Bloomberg Flow Cytometry and Immunology Core on a BD FACSymphony A5-SE spectral flow cytometer with FACS Diva software (version 9.0) and analyzed using FlowJo (version 10.10). Aberrant events were removed by the FlowAI plugin and gating based on fluorescence minus one (FMO). From live CD45^+^ cells, the populations were defined as outlined in Table S1.

### 2.10. Statistics

Statistical analyses were performed using GraphPad Prism 10. qRT-PCR, Western blotting, ELISA, and viral titer time course assays were analyzed for significant differences at each timepoint by two-way analysis of variance (ANOVA) with Bonferroni’s multiple comparisons post-test. Single-time point flow cytometry assays were analyzed by unpaired Student’s *t* test. Survival was assessed using a Kaplan–Meier curve and the log-rank test. A *p*-value of <0.05 was considered significant for all tests.

## 3. Results

**Figure 1:**
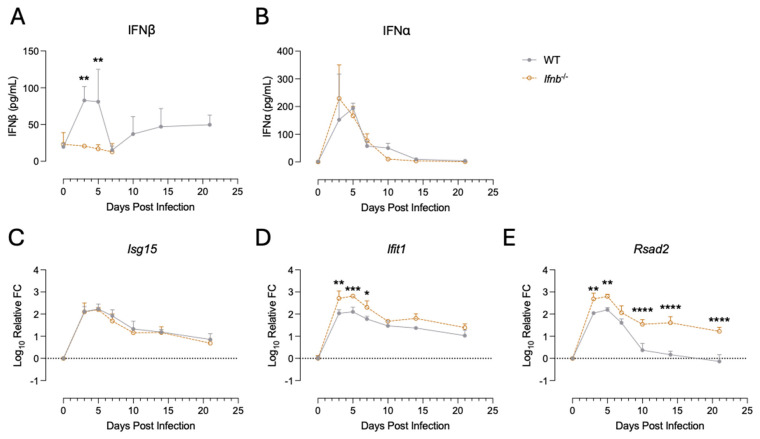
Type I IFN responses in the brains of WT and *Ifnb*^−/−^ mice. Type I IFN (**A**,**B**) and ISG (**C**–**E**) expression was assessed in the brains of mice at various time points after intracerebral SINV infection. IFN levels were measured by ELISA, and ISG levels by qRT-PCR. All data are presented as the mean ± SD from three or four individual mice per time point per genotype from two independent experiments. No indicator, non-significant, * *p* < 0.05, ** *p* < 0.01, *** *p* < 0.001, **** *p* < 0.0001; Bonferroni’s multiple comparisons test.

***Ifnb*^−/−^ mice retain normal IFNα expression.** IFNβ is one of the main members of the type I IFN family, an important and early acting class of antiviral cytokines. We intracerebrally infected WT and *Ifnb*^−/−^ mice with the TE strain of SINV, which causes neurological disease that WT mice can recover from [13], to assess the effects of IFNβ deficiency on the immune response and disease outcome. To determine whether loss of IFNβ expression altered levels of type I IFN, we measured IFN levels by ELISA (Figure 1A,B). IFNβ levels rapidly increased in the CNS of WT mice by 3 dpi, whereas IFNβ was undetectable in *Ifnb*^−/−^ mice following SINV infection (Figure 1A). Additionally, loss of IFNβ expression did not alter expression of IFNα, as both WT and *Ifnb*^−/−^ mice expressed IFNα to similar levels (Figure 1B).

In addition to IFN expression, we also examined the induction of ISGs, the major downstream effectors of type I IFN signaling. We used qRT-PCR to measure levels of *Isg15*, *Ifit1*, and *Rsad2* (Figure 1C–E), which play important roles in restricting SINV replication [59,60,61]. *Isg15* levels (Figure 1C) in *Ifnb*^−/−^ mice were identical to WT mice, and *Ifit1* and *Rsad2* mRNAs were significantly elevated in *Ifnb*^−/−^ mice compared to WT (Figure 1D,E). Taken together, despite a deficiency in one of the main type I IFNs, *Ifnb*^−/−^ mice still induced ISGs at or above the level of WT mice.

**Figure 2:**
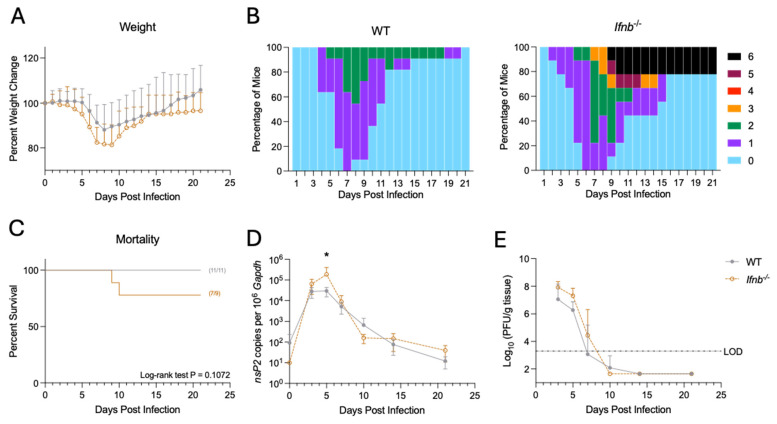
Disease manifestations and viral replication kinetics in WT and *Ifnb*^−/−^ mice. 5–8-week-old male and female WT or *Ifnb*^−/−^ mice were intracerebrally inoculated with 10^3^ PFU of SINV TE. Mice were monitored daily for signs of weight loss (**A**), clinical disease (**B**), and death (**C**), with *n* = 11 for WT mice and *n* = 9 for *Ifnb*^−/−^ mice. The clinical scoring system was: 0 = clinically normal; 1 = abnormal gait and/or ataxia; 2 = mild hunched posture with occasional two-leg stance but normal demeaner and ambulation; 3 = markedly hunched posture with minimal ambulation or obtunded; 4 = unilateral hindlimb paralysis; 5 = bilateral hindlimb paralysis or moribund; and 6 = death. Virus titers in the brains were assessed by qRT-PCR (**D**) and plaque formation (**E**). Data are presented as the mean ± SD from three to four individual mice per time point per genotype from three independent experiments. No indicator, non-significant, * *p* < 0.05; Bonferroni’s multiple comparisons test. Survival was assessed using a Kaplan–Meier curve and the log-rank test.

***Ifnb*^−/−^ mice show altered viral RNA replication and disease progression.** We next examined disease manifestation to determine whether loss of IFNβ altered clinical outcome following SINV infection (Figure 2A–C). IFNβ-deficient mice had slightly greater weight loss compared to WT mice, losing roughly 20% of initial body weight on average, compared to about 10% in WT mice (Figure 2A). For clinical disease, time to onset of symptoms occurred earlier in *Ifnb*^−/−^ mice, and a subset of *Ifnb*^−/−^ mice reached a clinical score of ‘5’, whereas no WT mice exceeded a clinical score of ‘2’ (Figure 2B). However, despite *Ifnb*^−/−^ mice demonstrating more severe disease at its peak, both genotypes recovered by approximately 15 dpi. Lastly, IFNβ-deficiency resulted in ~20% mortality compared to 0% mortality in WT mice, though the difference was not statistically significant (Figure 2C).

We also examined viral kinetics in the brains of WT and *Ifnb*^−/−^ mice after SINV infection (Figure 2D,E). IFNβ-deficient mice had higher amounts of viral RNA at 5 dpi, but the rate of clearance of viral RNA after that point paralleled that of WT mice (Figure 2D). Similarly, when measuring infectious virus, *Ifnb*^−/−^ mice had slightly elevated levels of virus at 3, 5, and 7 dpi, though the results were not statistically significant (Figure 2E). Therefore, IFNβ deficiency is correlated with increased viral RNA replication during peak infection but has little to no effect on the formation of infectious virus or the kinetics of viral clearance at later points during infection.

**Figure 3:**
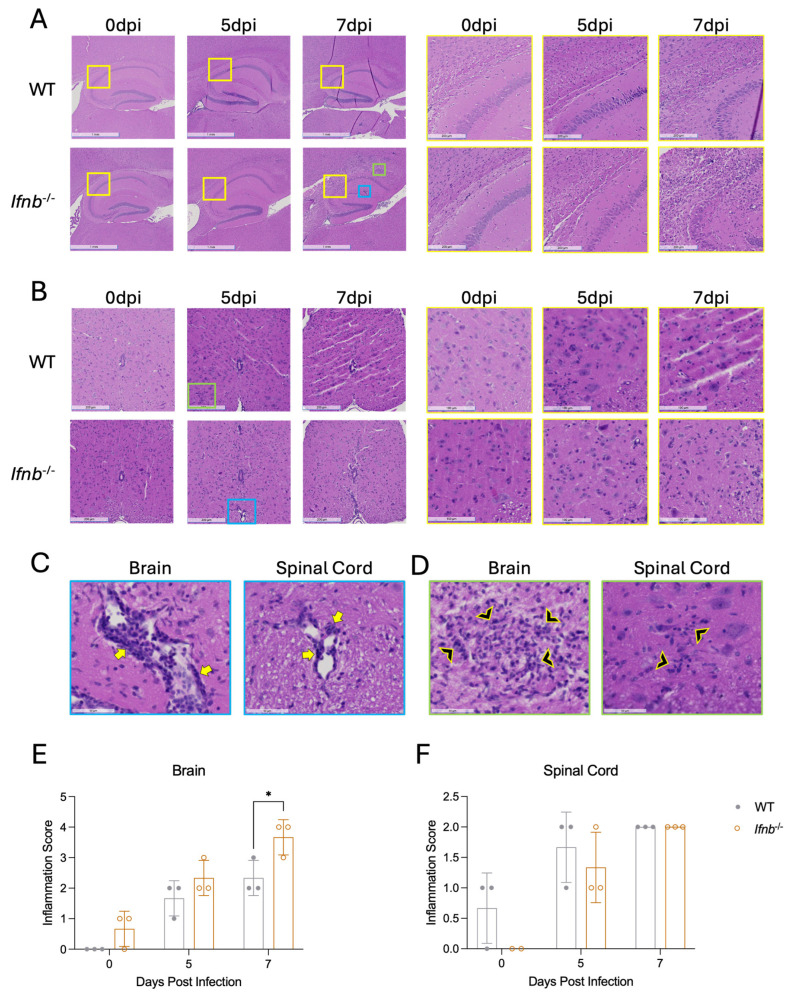
Brain and spinal cord pathology and inflammation after SINV infection of WT and *Ifnb*^−/−^ mice. The effect of IFNβ deficiency on pathology and CNS inflammation during SINV infection. (**A**,**B**) Representative photomicrographs of H&E-stained sections of the hippocampus (**A**) and lumbar spinal cord (**B**) of uninfected and infected (5 and 7 dpi) mice. Brains were scanned at 1× magnification (scale bar = 1 mm) with yellow insets scanned at 4× magnification (scale bar = 200 µm). Spinal cords were scanned at 4× magnification (scale bar = 200 µm) with yellow insets scanned at 10× magnification (scale bar = 100 µm). (**C**,**D**) Examples of perivascular cuffing ((**C**), yellow arrows, blue insets) and increased parenchymal cellularity ((**D**), black arrowheads, green insets) used for inflammatory scoring in brains (**left**) and spinal cords (**right**) at 20× magnification. (**E**,**F**) Sections were scored for inflammation using a 4-point ((**E**), brain) or 3-point ((**F**), spinal cord) system (see Methods). Each data point represents an individual mouse. Data are presented as the mean ± SD for three individual mice per time point per genotype from three independent experiments. No indicator, non-significant, * *p* < 0.05; Bonferroni’s multiple comparisons test.

**IFNβ deficiency alters brain and spinal cord pathology and inflammation after SINV infection.** To determine whether there were differences in histopathology between WT and *Ifnb*^−/−^ mice following SINV infection, sagittal brain (Figure 3A) and transverse lumbar spinal cord (Figure 3B) histological sections of uninfected and SINV-infected mice at 5 and 7 dpi were stained with H&E and evaluated for cellular and structural changes and evidence of inflammation. While inflammation and pathological changes were detectable throughout the brain, the corpus callosum and hippocampus (Figure 3A, yellow insets), whose neurons are targeted by SINV infection [62], were especially affected. Representative images of perivascular cuffing (Figure 3C, blue insets) and increased parenchymal cellularity (Figure 3D, green insets) at 20× magnification are shown, and both were evident by 5 dpi in the hippocampi and spinal cords of both WT and *Ifnb*^−/−^ mice (Figure 3A,B). At 7 dpi, pathological changes, specifically increased inflammatory cell infiltration, were noted in the corpus callosum of *Ifnb*^−/−^ mice that were absent from WT mice (Figure 3A). No histopathologic differences were detected in the 7 dpi spinal cords between genotypes (Figure 3B).

To quantify the level of inflammation, H&E-stained sections were blindly scored using a modified version of our previously described system [15]. Brain inflammation scores of *Ifnb*^−/−^ mice trended higher at 5 dpi and were significantly increased at 7 dpi (Figure 3E). Spinal cord inflammation scores did not differ between genotypes (Figure 3F). Taken together, IFNβ-deficient mice had increased pathological changes and inflammation in the brain compared to WT mice during SINV infection, indicating a role for IFNβ in regulating inflammation and pathology.

**Figure 4:**
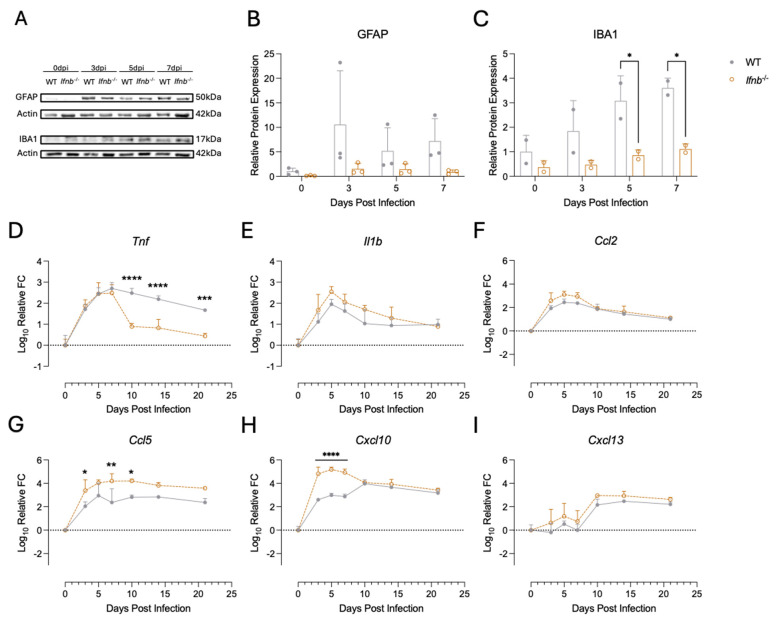
Innate inflammation in the brains of WT and *Ifnb*^−/−^ mice. Astrogliosis and microgliosis were assessed in the brains of uninfected (0 dpi) and SINV-infected mice at 3, 5, and 7 dpi. Western blotting for GFAP (**A**,**B**) and IBA1 (**A**,**C**) was performed on brain homogenates with actin used as a loading control. Data are expressed as the value of immunoreactivity of GFAP or IBA1 normalized to that of the uninfected WT mouse (**B**,**C**) from two to three independent experiments, and presented as mean ± SD. Expression of the cytokines *Tnf* (**D**) and *Il1b* (**E**), and the chemokines *Ccl2* (**F**), *Ccl5* (**G**), *Cxcl10* (**H**), and *Cxcl13* (**I**) in the brain was measured by qRT-PCR. Expression was normalized to *Gapdh* and compared to uninfected controls of each genotype. Data are presented as the mean ± SD for three individual mice per time point per genotype from two independent experiments. No indicator, non-significant, * *p* < 0.05, ** *p* < 0.01, *** *p* < 0.001, **** *p* < 0.0001; Bonferroni’s multiple comparisons test.

**Innate inflammation is altered in *Ifnb*^−/−^ mice**. Aside from the effects of IFNβ deficiency on clinical disease and virus replication, we wanted to determine how IFNβ production influences the nature of the inflammatory response to SINV infection in the brain. Proliferation and activation of microglia (gliosis) and astrocytes (astrogliosis) are a signature following CNS injury or infection [63,64,65]. We looked for signs of astrogliosis and microgliosis by blotting for GFAP and IBA1 [65], respectively (Figure 4A–C). In the brains of SINV-infected mice, GFAP levels trended lower in *Ifnb*^−/−^ mice over the course of infection (Figure 4A,B); however, these findings did not reach statistical significance. On the other hand, IBA1 levels were markedly reduced in *Ifnb*^−/−^ mice at 5 and 7 dpi (Figure 4A,C). These results indicate that IFNβ plays a role in regulating microglial, and possibly astrocyte, dynamics during SINV challenge.

We next looked at inflammatory cytokine and chemokine expression in the brain by qRT-PCR (Figure 4D–I). *Tnf* expression (Figure 4D) in the early stages of infection (0–7 dpi) was similar between WT and *Ifnb*^−/−^ mice, but was downregulated much faster in *Ifnb*^−/−^ mice at later time points (10–21 dpi). *Il1b* expression trended higher at varying times throughout infection in *Ifnb*^−/−^ mice but did not reach statistical significance (Figure 4E). Chemokine expression was also altered in the absence of IFNβ. Both *Ccl5* and *Cxcl10* were expressed at higher levels in *Ifnb*^−/−^ mice (Figure 4G,H) at the early time points during infection compared to WT mice, while expression of *Ccl2* and *Cxcl13* in *Ifnb*^−/−^ mice was unchanged compared to WT mice (Figure 4F,I). Altogether, we observed elevated expression of some inflammatory markers in the brains of *Ifnb*^−/−^ mice compared to WT mice following SINV infection, but this was not associated with microglial activation (Figure 4C), suggesting that inflammatory cytokine and chemokine expression could be derived from infiltrating leukocytes. Indeed, Cook et al. (2019) reported enhanced immune cell infiltration in the absence of INFβ during infection with a related alphavirus [49].

**Figure 5:**
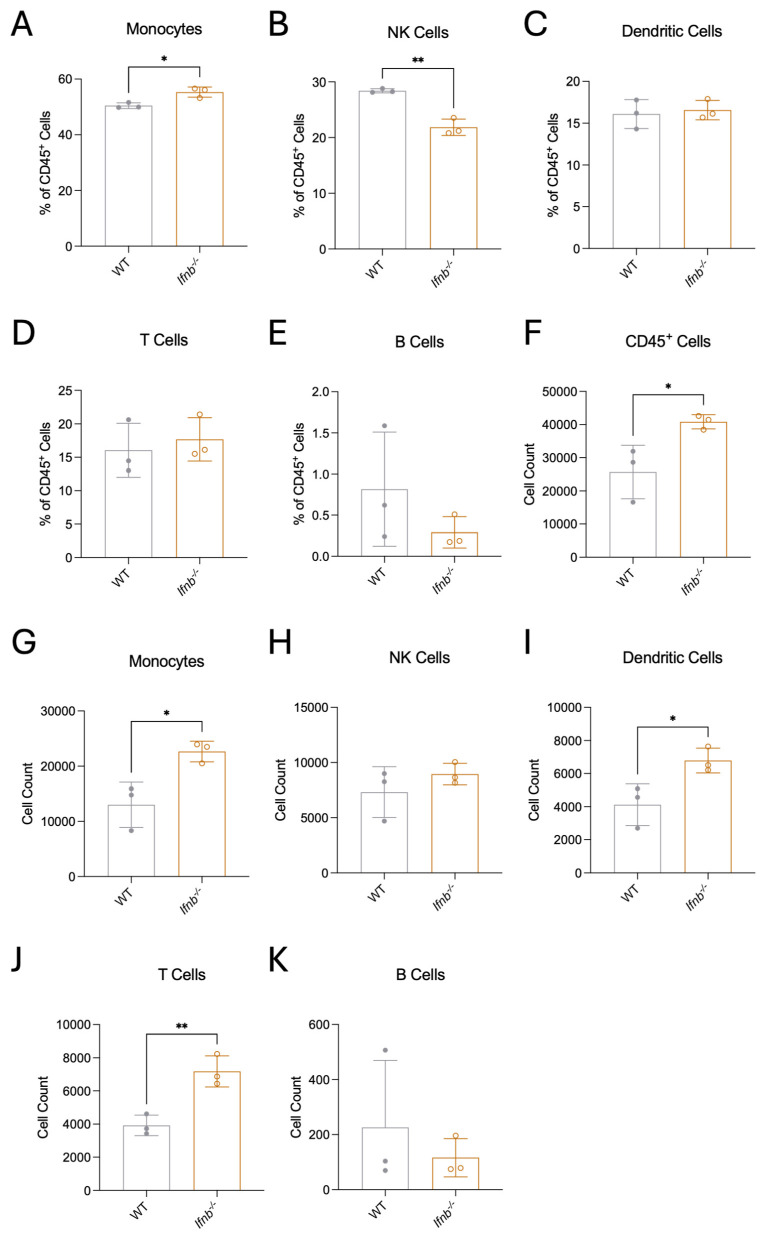
Immune cells infiltrate into the brain at 5 dpi. Flow cytometry analysis of infiltrating immune cells isolated from the brains of mice 5 days after infection with SINV. Percentages of CD45^+^ cells that were monocytes (**A**), natural killer (NK) cells (**B**), dendritic cells (DC) (**C**), T cells (**D**), or B cells (**E**), with corresponding cell counts (**F**–**K**). Cells were pooled from 5 to 7 mice per genotype for each data point, and the data are presented as mean ± SD from three independent experiments, and all samples were stained and assayed on the same day. No indicator, non-significant, * *p* < 0.05, ** *p* < 0.01; unpaired Students *t* test.

To study immune cell dynamics, we employed flow cytometry to assess changes in monocytes and macrophages, dendritic cells, NK cells, T cells, and B cells (Figure 5 and Figures S1–S3) infiltrating the brain at 5 dpi, the timepoint when nearly all mice were showing detectable signs of neurologic disease (Figure 2B) and peak IFN expression (Figure 1A,B). The analysis was restricted to CD45^hi^ cells (Table S1; Figure S1), to focus on infiltrating immune cells. We observed an increase in CD45^hi^ cell infiltration (Figure 5F) in *Ifnb*^−/−^ mice compared to WT, which is consistent with histopathologic findings (Figure 3E). While the proportion of monocytes was modestly elevated and NK cells were diminished in the CNS of *Ifnb*^−/−^ mice after SINV infection (Figure 5A,B), the proportions of DCs, T cells, B cells (Figure 5C–E), and their subsets (Figures S2 and S3) showed no significant changes between *Ifnb*^−/−^ and WT animals. The total number of cells infiltrating the brain for each population followed the same trends as the proportions (Figure 5G–K), with the exception of DCs (Figure 5I) and T cells (Figure 5J), which had increased numbers but not higher proportions (Figure 5C,D). This indicates that the increased CD45^hi^ infiltrate is primarily driven by increased monocyte, DC, and T cell populations, but only monocytes are overrepresented in *Ifnb*^−/−^ mice compared to WT.

**Figure 6:**
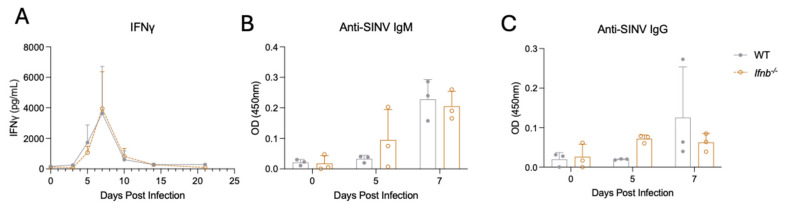
IFNγ and virus-specific antibody levels in the brains of WT and *Ifnb*^−/−^ mice. IFNγ (**A**) and virus-specific IgM (**B**) and IgG (**C**) levels were measured by ELISA. Data are presented as the mean ± SD from three to four individual mice per time point per genotype from three independent experiments. No indicator, non-significant; Bonferroni’s multiple comparisons test.

***Ifnb*^−/−^ mice express factors needed for viral clearance.** IFNγ and virus-specific antibodies are crucial mediators of SINV clearance from the CNS [16,56,66,67,68,69]. Therefore, we measured IFNγ and antibody levels in the brains of WT and *Ifnb*^−/−^ mice (Figure 6). IFNγ expression was identical over the course of infection between genotypes (Figure 6A). Virus-specific IgM (Figure 6B) and IgG (Figure 6C) titers were also similar across conditions during the early time points after infection. Because *Ifnb*^−/−^ mice do not differ from WT mice with regard to the production of factors known to be required for SINV clearance, it suggests that disparate disease phenotypes observed were caused by a lack of IFNβ signaling itself as opposed to the inability to express IFNγ, antibody, or the ISG effectors downstream of IFNβ signaling (Figure 1).

## 4. Discussion

Type I IFN is critical for recovery from peripheral infection with SINV [59,70,71,72,73], but its role in SINV-induced CNS disease has not been extensively studied, and type-specific functions have not been examined. As such, these studies aimed to characterize the specific impact of IFNβ on host outcome from SINV-induced encephalomyelitis by using *Ifnb*^−/−^ mice. These mice have intact IFNα expression and downstream ISG induction, thus providing a useful tool for studying the roles of IFNβ that is uncoupled from the actions of other type I IFNs. *Ifnb*^−/−^ mice have an earlier onset of neurologic disease that is slightly more severe in nature than in WT mice, and this phenotype is associated with increased peak viral titers in the brain, an elevated cellular infiltrate, and pathological changes. Changes to disease manifestation are also associated with alterations to innate immunity. *Ifnb*^−/−^ mice have lower amounts of astro- and microgliosis but higher inflammatory cytokine and chemokine expression than WT mice. Changes in immune cell trafficking into the CNS are also observed, with higher monocyte and lower NK cell proportions. Therefore, the findings indicate that IFNβ plays a role in early restriction of SINV replication and regulation of the innate immune response.

Per its namesake, interference with virus replication is the classical role for type I IFN. Therefore, it is unsurprising that *Ifnb*^−/−^ mice had heightened viral RNA replication compared to WT mice (Figure 2D,E). Our findings are consistent with previous work demonstrating higher titers of infectious virus in both the brains and spinal cords of *Ifnb*^−/−^ mice compared to WT mice at 3 dpi [56]. Additionally, studies with *Irf7*^−/−^ mice, widely used as a surrogate for IFNα-deficiency, have shown sustained virus replication and cell death in the absence of IFNα [19]. While a few studies have examined the effects of IFNβ- or IFNAR-deficiency on disease manifestation following SINV infection in the CNS, they fall short of a comprehensive characterization of the immune response [19,56,74].

Here, we observed the same or higher levels of ISG expression in *Ifnb*^−/−^ mice compared to WT controls (Figure 1). We expected a decrease in ISG expression due to the loss of IFNβ expression. As such, we speculate that the initial effect of IFNβ deficiency could be driven by altered expression of effector ISGs not examined here. Additionally, the sustained expression of *Rsad2* in the absence of IFNβ (Figure 1B) suggests an altered feedback loop that may affect immune-related genes associated with elevated *Rsad2*, possibly through compensation by IFNα or type III IFN. However, IFNβ’s ultimate impact on disease outcome appears to be driven by dysregulation of the immune response at later timepoints. This theory is supported by heightened viral RNA replication only being seen at 5 dpi (Figure 2D), which precedes peak disease occurring between 7 and 9 dpi (Figure 2B). SINV is unique in that viral titer does not necessarily correlate with disease severity, as SCID mice show mild but persistent infection and disease following SINV infection [75]. Furthermore, the arrival of peripheral lymphocytes into the CNS, which are needed for SINV clearance, occurs concomitantly with disease onset [15,69,76]. Therefore, defining the impact of IFNβ deficiency on the immune response is crucial to understanding the pathogenesis during SINV infection of *Ifnb*^−/−^ mice.

We found that *Ifnb*^−/−^ mice have increased pathological changes and inflammation (Figure 3A,C) associated with lowered gliosis after SINV infection compared to WT controls (Figure 4A–C). Astrocytes and microglia are very strong responders to type I IFN, and IFN signaling is important for their activation and polarization towards an antiviral state [77,78,79,80]. Therefore, lower levels of reactivity from first responder resident CNS immune cells in *Ifnb*^−/−^ mice could negatively affect early responses to viral infection. One downstream result of this phenomenon could be an impaired restriction of virus replication, supported by higher viral RNA levels in *Ifnb*^−/−^ mice at 5 dpi (Figure 2D). Other studies have shown that microglia are important sources of type I IFN and inflammatory cytokines during SINV infection [81]. While *Ifnb*^−/−^ mice did not have major deficits in expression of the select cytokines and chemokines measured here, it is possible that differences driven by gliosis happened earlier than 3 dpi, the first time point examined (Figure 4). Furthermore, the interpretation of cytokine expression at later time points was confounded by the infiltration of monocytes, which are also important sources of the same inflammatory factors. Therefore, deficits in gliosis early on could have resulted in the higher viral RNA we observed at 5 dpi. Interestingly, previous studies in *Irf7*^−/−^ mice show no differences in gliosis compared to WT, which suggests that gliosis is impacted by IFNβ deficiency but not IFNα deficiency [18].

Of note, chemokine expression in the brain was higher in IFNβ-deficient mice compared to WT mice, particularly for *Ccl5* and *Cxcl10* (Figure 4). These are broad inflammatory chemokines that attract a wide range of cells [82,83,84], which could explain the increased monocytes in the brains of *Ifnb*^−/−^ mice (Figure 5A). The roles ascribed to monocytes during viral infection are often paradoxical, as they are implicated in driving pathogenesis while also facilitating viral clearance through activation of adaptive immune responses [85]. However, in the case of alphavirus infection, monocytes tend to primarily have beneficial roles. For example, depletion of Ly6C^+^ monocytes during CHIKV and RRV infections results in increased viral load and virulence, and ablation of myeloid cells following infection with a highly neurovirulent strain of SINV (NSV) caused more rapid death. The exact contribution of monocytes to the pathogenesis of *Ifnb*^−/−^ mice during SINV TE infection is the focus of ongoing studies, including the contribution of reduced microgliosis. Additionally, alterations to chemokine expression likely account for the reduced NK cell influx into the brain (Figure 5B). NK cells are important producers of IFNγ and CCL5 during SINV infection [86,87]; therefore, reductions in NK cells could impact the early restriction of virus replication and immune cell recruitment into the CNS. However, overall levels of IFNγ and *Ccl5* in the brains of these mice were not different from those of WT mice, and NK cells contribute minimally toward recovery from SINV infection [88]; therefore, impactful consequences from this reduction are unlikely.

Taken together, the role type I IFN plays during viral infection is extremely important, underscored by the fact that alphaviruses have evolved ways of suppressing IFN [73,89,90]. These studies have demonstrated a role for IFNβ in early restriction of virus replication and modulation of the innate immune response. Whether these changes to viral replication and the immune response are linked remains unclear, but both likely contribute toward disease manifestation. While the focus of this study looked at early stages in infection and innate immune response, it is also of interest to examine the effects of IFNβ deficiency on the adaptive response, as IFN modulates T and B cell responses as well [20,37,38,91,92,93,94]. Further understanding of how type I IFN affects virus replication and the immune response, the interplay between the two, and any temporal differences can lead to potential treatment options for alphavirus encephalomyelitis.

## Data Availability

The original contributions presented in this study are included in the article/Supplementary Materials. Further inquiries can be directed to the corresponding author.

## Supplementary Materials

The following supporting information can be downloaded at https://www.mdpi.com/article/10.3390/pathogens15040441/s1, Table S1: Cell population definitions for flow cytometry. Defining markers used to identify cell types profiled by flow cytometry; Table S2: Antibodies used for flow cytometry; Figure S1: Flow cytometry gating. Flow cytometry gating strategy for all cell populations; Figure S2: Myeloid cell subset infiltrate into the brain at 5 dpi. Flow cytometry analysis of infiltrating myeloid and dendritic cell subsets isolated from the brains of mice 5 days after infection with SINV. Percentages of CD45^+^ cells that were classical monocytes (A), non-classical monocytes (B), intermediate monocytes (C), classical DC 1 (D), classical DC 2 (E), or plasmacytoid DCs (F) with corresponding cell counts (G–L). Cells were pooled from 5 to 7 mice per genotype for each data point, and the data are presented as mean ± SD. No indicator, non-significant, * *p* < 0.05, ** *p* < 0.01, *** *p* < 0.001, **** *p* < 0.0001; unpaired Student’s *t*-test; Figure S3: Lymphocyte subset infiltrate into the brain at 5 dpi. Flow cytometry analysis of infiltrating lymphoid cells isolated from the brains of mice 5 days after infection with SINV. Percentages of CD45^+^ cells that were CD8 T cells (A), CD4 T cells (B), B1 cells (C), B1a cells (D), or B1b cells (E) with corresponding cell counts (F–J). Cells were pooled from 5 to 7 mice per genotype for each data point, and the data are presented as mean ± SD. No indicator, non-significant, * *p* < 0.05, ** *p* < 0.01, *** *p* < 0.001, **** *p* < 0.0001; unpaired Student’s *t* test.
