# Supplementary material for: Interferon-β Modulates Early Viral Replication Kinetics and Innate Responses to Non-Fatal Alphavirus Encephalomyelitis
Source: Pathogens. 2026 Apr 18;15(4):441. doi: 10.3390/pathogens15040441 (PMC13119104; doi:10.3390/pathogens15040441)
Supplement: Supplementary file 1 [file pathogens-15-00441-s001.zip › pathogens-4197963-supplementary.pdf]

Table S1

Cell Type	Markers
T cells	$CD3^{+}$
CD8 T cells	$CD3^{+}$ $CD4^{-}$ $CD8^{+}$
CD4 T cells	$CD3^{+}$ $CD4^{+}$ $CD8^{-}$
NK Cells	$CD3^{-}$ $NK1.1^{+}$
B cells	$CD19^{+}$
Dendritic cells	$CD11c^{+}$ $MHC\text{-}II^{+}$
Common DC 1	$CD11c^{+}$ $MHC\text{-}II^{+}$ $CD11b^{-}$ $CD103^{+}$
Common DC 2	$CD11c^{+}$ $MHC\text{-}II^{+}$ $CD11b^{+}$ $CD103^{-}$
Plasmacytoid DC	$CD11c^{+}$ $MHC\text{-}II^{-}$ $SiglecH^{+}$
Monocytes	$CD11b^{+}$ $F4/80^{-}$
Classical monocytes	$CD11b^{+}$ $F4/80^{-}$ $Ly6C^{+}$ $CD43^{-}$
Non-classical monocytes	$CD11b^{+}$ $F4/80^{-}$ $Ly6C^{-}$ $CD43^{+}$
Intermediate monocytes	$CD11b^{+}$ $F4/80^{-}$ $Ly6C^{+}$ $CD43^{+}$

Table S2

Target	Fluorophore	Catalog #	Vendor
CD45	Pacific Blue™	103125	BioLegend
CD11b	Brilliant Violet 570™	101233	BioLegend
F4/80	Spark YG™ 593	157311	BioLegend
MHC II	PerCP/Fire™ 806	107673	BioLegend
Ly6C	Brilliant Violet 650™	128049	BioLegend
CD43	BD Horizon™ BUV737	612840	BD
CD11c	Spark NIR™ 685	117367	BioLegend
CD103	Brilliant Violet 605™	121433	BioLegend
SiglecH	APC	129611	BioLegend
CD3	Spark UV™ 387	100283	BioLegend
CD3	BD Horizon™ BUV395	569614	BioLegend
CD4	Spark Blue™ 550	100473	BioLegend
CD8	Brilliant Violet 510™	100751	BioLegend
NK1.1	PE/Fire™ 700	156527	BioLegend
CD19	Spark Blue™ 574	115581	BioLegend
L/D Near-IR	Near-IR 633	L10119	BioLegend
Ly6G	APC/Fire™ 750	127651	BioLegend

Figure S1

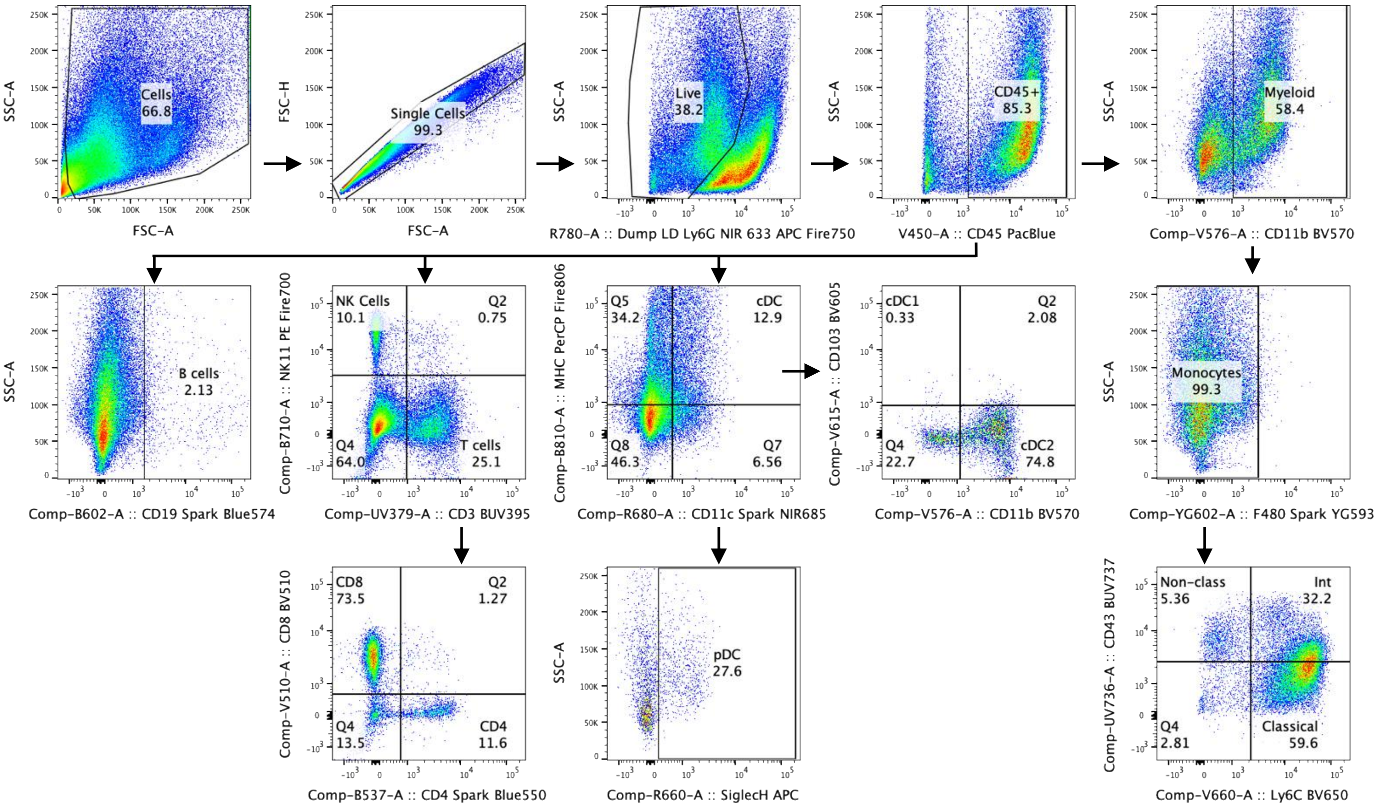

Figure S1. Representative flow cytometry gating scheme

Figure S2

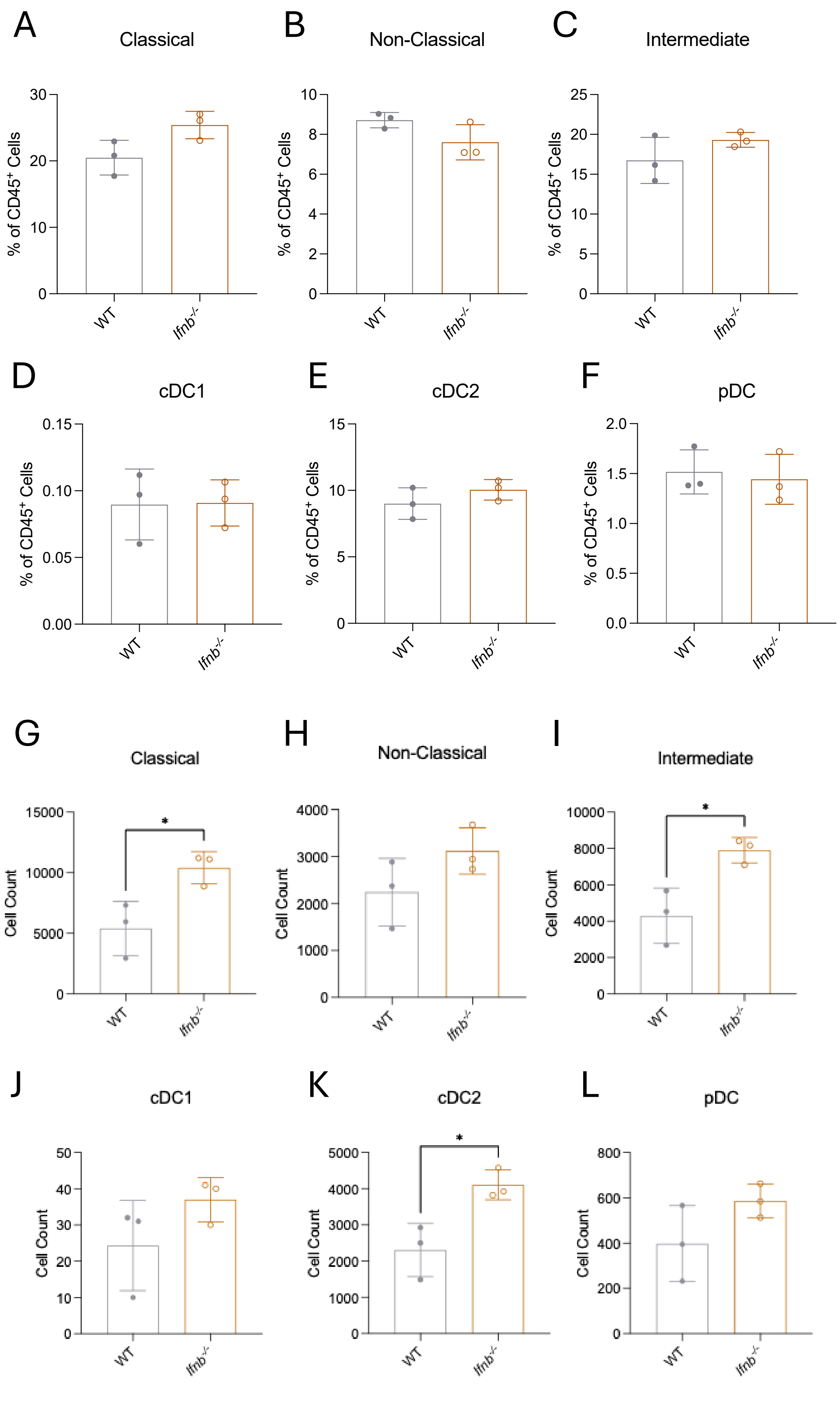

Figure S2. Innate immune cell infiltrate into the brain at 5dpi. Flow cytometry analysis of infiltrating immune cells isolated from the brains of mice 5 days after infection with SINV. Percentages of CD45+ cells that were classical (A), non-classical (B), or intermediate (C) monocytes, cDC1 (D), cDC2 (E), or pDC (F) with corresponding cell counts (G-L). Cells were pooled from 5-7 mice per genotype for each data point, and the data are presented as mean ± SD from three independent experiments and all samples were stained and flowed on the same day. No indicator, non-significant, *P < 0.05, **P < 0.01, ***P < 0.001, ****P < 0.0001; unpaired Students t test.

Figure S3

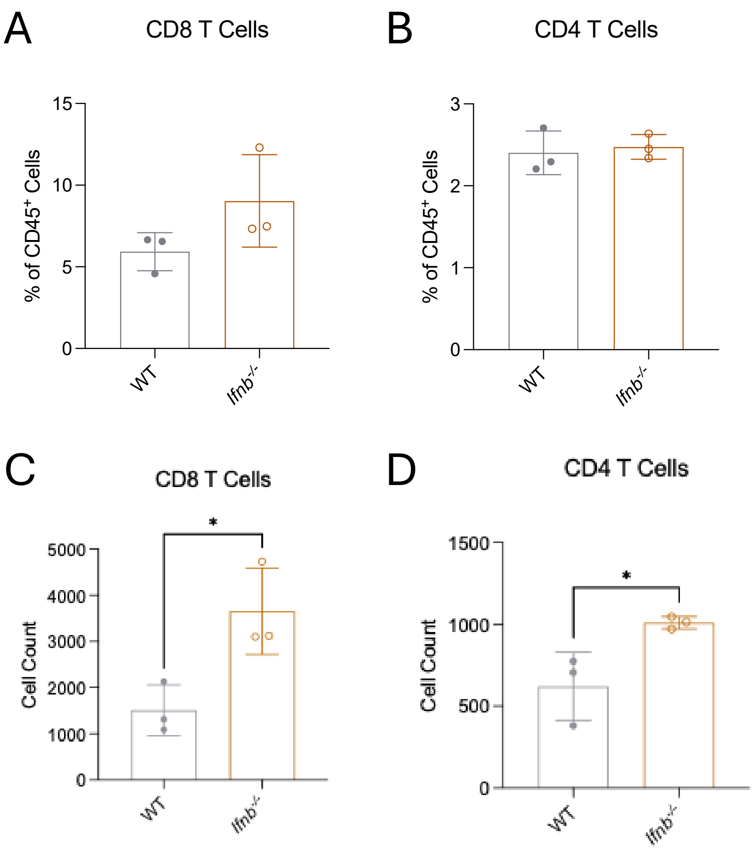

Figure S3. Adaptive immune cell infiltrate into the brain at 5dpi. Flow cytometry analysis of infiltrating immune cells isolated from the brains of mice 5 days after infection with SINV. Percentages of CD45+ cells that were CD8 (A) or CD4 (B) T cells with corresponding cell counts (C,D). Cells were pooled from 5-7 mice per genotype for each data point, and the data are presented as mean ± SD from three independent experiments and all samples were stained and flowed on the same day. No indicator, non-significant, *P < 0.05, **P < 0.01, ***P < 0.001, ****P < 0.0001; unpaired Students t test.
